# Small bowel volvulus caused by a congenital band in a young adult: indirect computed tomography signs and surgical correlation

**DOI:** 10.1093/jscr/rjag785

**Published:** 2026-09-09

**Authors:** Omar Ibelkouchene, Ayoub Ajerame, Ola Messaoud, Omar El Aoufir, Laila Jroundi, Zaynab Iraqi Houssaini

**Affiliations:** Emergency Radiology Department, Ibn Sina Hospital, Mohammed V University, 10100 Rabat, Morroco; Emergency Radiology Department, Ibn Sina Hospital, Mohammed V University, 10100 Rabat, Morroco; Emergency Radiology Department, Ibn Sina Hospital, Mohammed V University, 10100 Rabat, Morroco; Emergency Radiology Department, Ibn Sina Hospital, Mohammed V University, 10100 Rabat, Morroco; Emergency Radiology Department, Ibn Sina Hospital, Mohammed V University, 10100 Rabat, Morroco; Emergency Radiology Department, Ibn Sina Hospital, Mohammed V University, 10100 Rabat, Morroco

**Keywords:** small bowel obstruction, congenital band, small-bowel volvulus, computed tomography, beak sign, fatty notch sign

## Abstract

Small-bowel volvulus caused by a congenital band is an uncommon etiology of small-bowel obstruction in adults, particularly in young patients without prior abdominal surgery. We report the case of a 20-year-old woman presenting with acute small-bowel obstruction. Contrast-enhanced computed tomography (CT) demonstrated dilated proximal bowel loops with a focal transition point, associated with a beak sign, fatty notch sign, and mesenteric whirl sign, suggestive of volvulus due to a single obstructing band, without signs of bowel ischemia. Surgical exploration confirmed a congenital band between two ileal loops, which was successfully divided with an uneventful postoperative outcome. This case highlights the diagnostic value of recognizing specific CT features that may suggest congenital bands and facilitate timely surgical management.

## Introduction

Small bowel obstruction is a frequent cause of emergency department admission and accounts for a significant proportion of acute abdominal pain. In adults, postoperative adhesions represent the most common etiology, while congenital bands are a rare cause, particularly in patients with no history of abdominal surgery.

Although congenital bands are uncommon, a delayed diagnosis may result in severe and potentially life-threatening complications. These bands arise from abnormal peritoneal adhesions formed during embryogenesis and may be located at various levels of the gastrointestinal tract, including the ileum, ascending colon, or the ligament of Treitz. Consequently, congenital bands should be considered in cases of atypical presentation of small bowel obstruction, even in the absence of previous abdominal surgery or trauma.

Computed tomography (CT) is the imaging modality of choice for evaluating suspected small bowel obstruction, as it allows accurate identification of the level of obstruction, the underlying cause, and potential complications.

We report a case of small bowel obstruction caused by a congenital band in a young adult, highlighting the diagnostic value of CT findings and their correlation with surgical management.

## Case report

A 20-year-old female patient with no history of abdominopelvic surgery, inflammatory bowel disease, or trauma presented for a four days history of diffuse abdominal pain, mainly at the right iliac fossa pain. The pain was associated with vomiting and obstipation of two days duration.

Upon physical exam, the patient appeared pale, with a distended, soft abdomen, and positive bowel sounds. Right iliac fossa tenderness was noted on palpation.

Vital signs were as follows: heart rate 85, O_2_ saturation 99%, respiratory rate 20, temperature 37°C.

Baseline laboratory tests, including a complete blood count, serum electrolytes, and inflammatory markers, were not significant.

**Figure 1:**
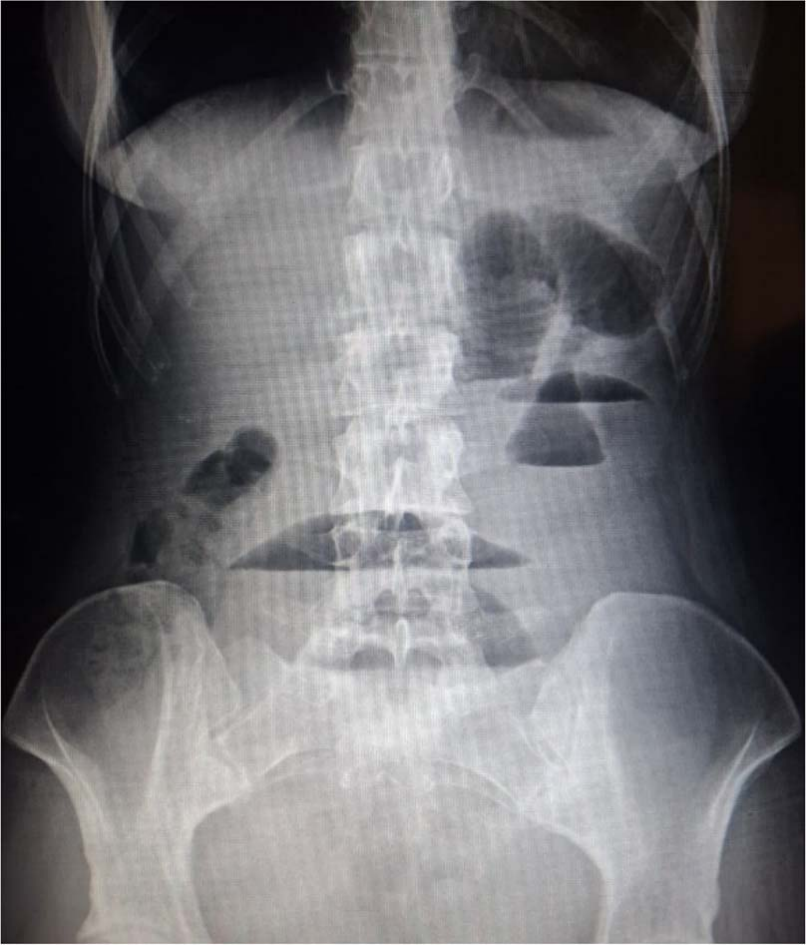
Plain abdominal radiograph showing centrally distributed air–fluid levels, wider than tall, consistent with small-bowel obstruction.

Plain radiography of the abdomen demonstrated centrally distributed air–fluid levels that were wider than tall, suggestive of a small-bowel obstruction (Fig. 1).

**Figure 2:**
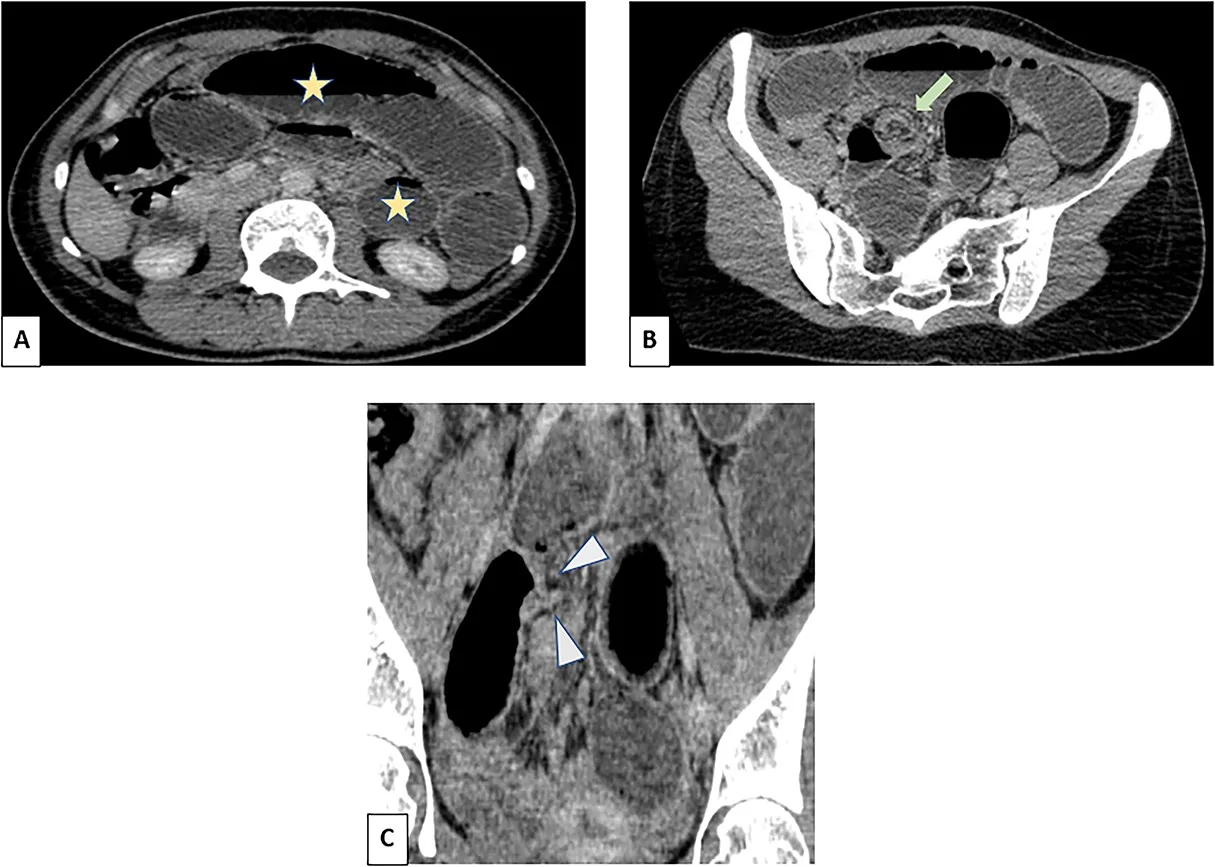
Contrast-enhanced abdominal CT showing dilated small-bowel loops with air–fluid levels (asterisks) proximal to a focal transition point forming the beak sign (arrow). Twisting of the bowel loops and mesenteric vessels produces the whirl sign (arrowhead). At the same level, a focal extraluminal fatty indentation corresponding to the fatty notch sign is identified, reflecting mesenteric fat insinuation at the transition point.

Contrast-enhanced CT demonstrated a disparity of intestinal caliber, with dilated small bowel loops measured 40 mm in maximum diameter and flat loops with a single transitional zone in the right pelvic region. The transition zone demonstrated a beak sign, associated with a fatty notch sign, and a twisting of the bowel and mesenteric vessels (whirl sign), consistent with small-bowel volvulus. No CT signs of bowel ischemia were identified (Fig. 2).

**Figure 3:**
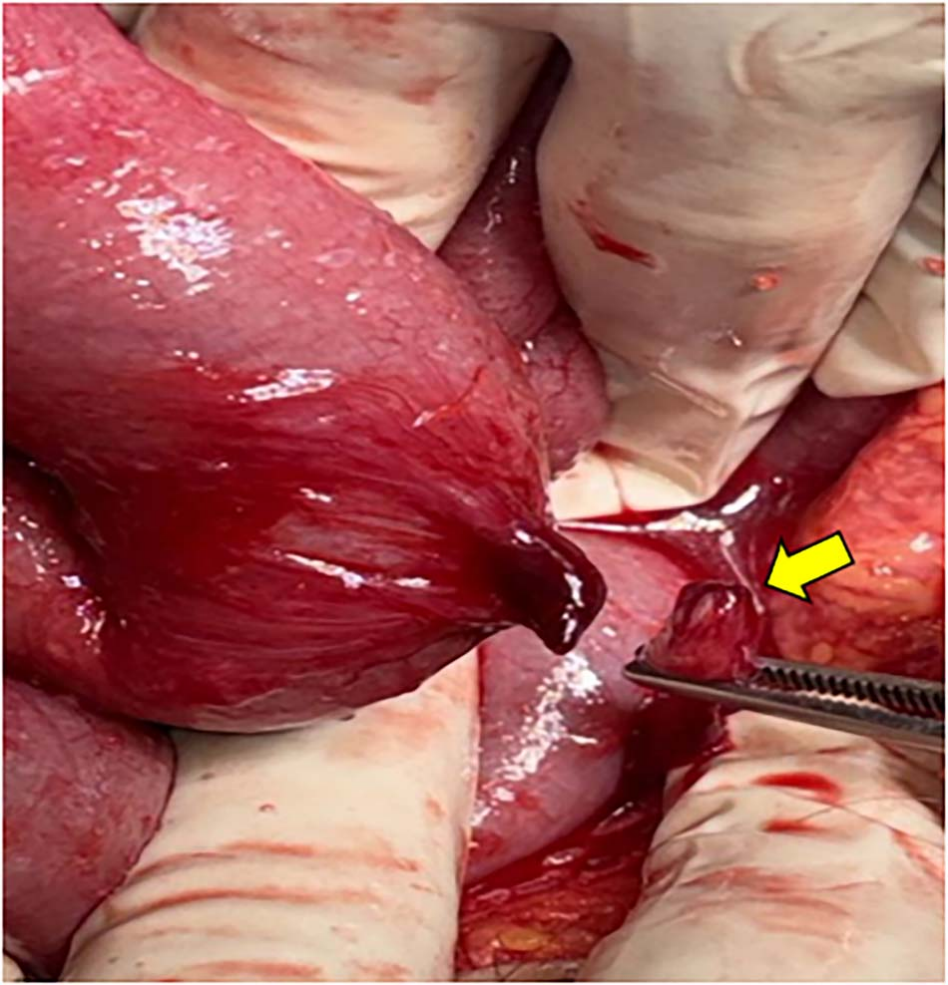
Intraoperative photograph showing a congenital band between two ileal loops, responsible for the small-bowel obstruction. The band was surgically divided, with no evidence of bowel ischemia or necrosis.

A laparotomy was performed, confirming a congenital band located between two ileal loops (Fig. 3) that was responsible for the obstruction. There were no signs of ischemia or necrosis. The band was ligated and divided, and the postoperative course was uneventful.

## Discussion

Small bowel volvulus secondary to a congenital band remains an exceptionally rare cause of small bowel obstruction in adults [1, 2], particularly in young patients with no prior history of abdominal surgery. Congenital bands account for ~2%–6% of band-related small bowel obstructions, with the vast majority of cases reported in pediatric populations rather than adults [3].

In a retrospective review of 251 patients with small bowel obstruction, 15 cases (5.9%) were caused by congenital bands, with only a minority occurring in adults, emphasizing the rarity of this diagnosis in the adult age group [3]. Adult surgical series, including cohorts of ~16 patients, consistently report the absence of prior abdominal surgery and a variable risk of bowel ischemia, highlighting the diagnostic challenge of this entity [4].

From an embryologic perspective, congenital bands are believed to arise from the abnormal persistence or fusion of peritoneal folds during midgut development. Remnants of embryonic structures such as the vitelline duct, vitelline vessels, or anomalous mesenteric attachments may form fibrous bands that remain asymptomatic until they cause mechanical obstruction or serve as a pivot point for volvulus [5].

Most adult cases reported in the literature share clinical features similar to those observed in our patient, including young age, absence of surgical history, and nonspecific obstructive symptoms such as abdominal pain, vomiting, and a cessation of stool and gas [2, 6]. Recent case reports continue to emphasize the role of CT in suggesting the diagnosis, even when direct visualization of the band is not possible [6, 7].

Direct visualization of a congenital band on CT remains exceptional, although it may occasionally appear as a thin, elongated soft-tissue structure when image quality permits [7]. Consequently, diagnosis most often relies on indirect but increasingly recognized positive CT signs. The beak sign, representing abrupt luminal narrowing at the transition point, has been described for more than two decades and is characteristic of an obstruction caused by a single adhesive band [8]. More recently, the fatty notch sign, corresponding to focal extraluminal compression of the bowel by the band, has been described as an additional helpful indicator [9].

Importantly, CT studies have demonstrated that small bowel obstruction caused by a single obstructing band differs from an obstruction due to matted adhesions, as it is more frequently associated with a sharp focal transition point, closed-loop obstruction, and volvulus, all of which carry a higher risk of bowel ischemia and favor early surgical management [8, 10]. Although initially described in postoperative adhesive small bowel obstruction, these radiologic principles apply equally to congenital bands, which behave as a fixed obstructing structure.

Features such as localized mesenteric twisting, reduced bowel wall enhancement, mesenteric edema, and peritoneal fluid raise concern for ischemia and necessitate prompt operative management [10].

The choice of surgical approach should be individualized according to the patient’s clinical status, preoperative CT findings, and the surgeon’s expertise. Although laparotomy remains the preferred approach in patients with a suspected volvulus, marked bowel dilatation, or a concern for bowel ischemia, laparoscopy has emerged as a safe and effective alternative in carefully selected patients. In hemodynamically stable individuals without evidence of bowel ischemia, perforation, or severe bowel distension, laparoscopic division of a congenital band may offer the advantages of reduced postoperative pain, shorter hospital stay, and faster recovery [11, 12].

In the present case, the CT findings of a small bowel volvulus with significant bowel dilatation prompted an open surgical approach. Laparotomy confirmed a congenital band between two ileal loops without bowel ischemia, allowing safe division of the band with an uneventful postoperative course.

Our report aligns with these rare adult presentations and reinforces the need to consider congenital bands in the differential diagnosis of small bowel obstruction in young adults. Early recognition of subtle CT features can facilitate timely surgical intervention and improve patient outcomes.

## Conclusion

Congenital bands are an uncommon but important cause of small-bowel obstruction in young adults without prior abdominal surgery. Because the band itself is rarely seen on CT, diagnosis relies on recognition of key indirect signs, including a sharp transition point, beak sign, fatty notch sign, and occasionally a whirl sign indicating volvulus. Early detection of these features facilitates prompt surgical intervention, reduces the risk of bowel ischemia, and improves outcomes. Radiologists should be aware of these subtle findings to guide timely management in this rare but potentially serious condition.

## References

[1] Ten Broek RPG, Krielen P, Di Saverio S et al. Adhesive small bowel obstruction: epidemiology, diagnosis and treatment. Nat Rev Gastroenterol Hepatol 2013;10:59–70.

[2] Akgur FM, Tanyel FC, Buyukpamukcu N et al. Congenital bands causing intestinal obstruction in children. J Pediatr Surg 1992;27:471–3. 10.1016/0022-3468(92)90340-D 1522460

[3] Di Saverio S, Catena F, Ansaloni L et al. Congenital bands: an uncommon cause of small bowel obstruction in adults. World J Gastroenterol 2012;18:6077–81.

[4] Miller G, Boman J, Shrier I, et al. Natural history of patients with adhesive small bowel obstruction. Br J Surg 2000;87:1240–7. 10.1046/j.1365-2168.2000.01530.x 10971435

[5] Moore KL, Persaud TVN. The Developing Human: Clinically Oriented Embryology, 8th edn. Philadelphia: Saunders, 2008.

[6] Habib E, Elhadad A, Arnaud JP et al. Small bowel obstruction due to congenital bands in adults: report of two cases. Surg Radiol Anat 2003;25:425–8.

[7] Sato M, Yamamoto T, Takahashi K et al. Small bowel obstruction due to congenital band in an adult: radiologic–surgical correlation. Radiol Case Rep 2019;14:1352–6.31516651

[8] Balthazar EJ, Liebowitz RL, Megibow AJ et al. CT of small-bowel obstruction: value in establishing the diagnosis and determining the degree and cause. AJR Am J Roentgenol 1997;168:1171–80. 9129407 10.2214/ajr.168.5.9129407

[9] Delabrousse E, Lubrano J, Jehl J, et al. Small-bowel obstruction from adhesive bands and matted adhesions: CT differentiation. AJR Am J Roentgenol 2009;192:693–7. 10.2214/AJR.08.1550 19234265

[10] Furukawa A, Yamasaki M, Furuichi K et al. CT diagnosis of small bowel obstruction: scanning technique, interpretation and role in management. Radiographics 2009;29:1819–35.

[11] Wu JM, Lin HF, Chen KH, et al. Laparoscopic diagnosis and treatment of acute small bowel obstruction resulting from a congenital band. Surg Laparosc Endosc Percutan Tech 2005;15:294–6. 16215491 10.1097/01.sle.0000183258.34926.59

[12] Atri S, Hammami M, Sebai A, et al. Congenital band as a rare cause of small bowel obstruction: a case series and review of the literature. World J Emerg Surg 2026;21:48. 10.1186/s13017-026-00704-z. PMC13474849 42260583

